# Clinical utility of testing for *PALB2* and *CHEK2* c.1100delC in breast and ovarian cancer

**DOI:** 10.1038/s41436-021-01234-6

**Published:** 2021-06-10

**Authors:** Emma R. Woodward, Elke M. van Veen, Claire Forde, Elaine F. Harkness, Helen J. Byers, Jamie M. Ellingford, George J. Burghel, Helene Schlech, Naomi L. Bowers, Andrew J. Wallace, Sacha J. Howell, Anthony Howell, Fiona Lalloo, William G. Newman, Miriam J. Smith, D. Gareth Evans

**Affiliations:** 1 Manchester Centre for Genomic Medicine, Manchester University Hospitals NHS Foundation Trust, Manchester, UK; 2 Division of Evolution and Genomic Sciences, School of Biological Sciences, Faculty of Biology, Medicine and Health, University of Manchester, Manchester Academic Health Science Centre, Manchester, UK; 3 Prevent Breast Cancer Centre, Wythenshawe Hospital Manchester Universities Foundation Trust, Wythenshawe, Manchester, UK; 4 Division of Informatics, Imaging and Data Sciences, Faculty of Biology, Medicine and Health, University of Manchester, Manchester Academic Health Science Centre, Manchester, UK; 5 Manchester Breast Centre, The Christie NHS Foundation Trust, Wilmslow Road, Manchester, UK; 6 Division of Cancer Sciences, Faculty of Biology, Medicine and Health, University of Manchester, Manchester Academic Health Science Centre, Manchester, UK

## Abstract

**Purpose:**

To investigate the contribution of *PALB2* pathogenic gene variants (PGVs, *PALB2*_PGV) and the *CHEK2* c.1100delC (*CHEK2*_1100delC) PGV to familial breast and ovarian cancer, and *PALB2*_PGV associated breast cancer pathology.

**Methods:**

Outcomes of germline *PALB2*_PGV and *CHEK2_*1100delC testing were recorded in 3,127 women with histologically confirmed diagnoses of invasive breast cancer, carcinoma in situ, or epithelial nonmucinous ovarian cancer, and 1,567 female controls. Breast cancer pathology was recorded in *PALB2*_PGV cases from extended families.

**Results:**

Thirty-five *PALB2* and 44 *CHEK2*_1100delC PGVs were detected in patients (odds ratio [OR] *PALB2* breast–ovarian = 5.90 [95% CI: 1.92–18.36], *CHEK2* breast–ovarian = 4.46 [95% CI: 1.86–10.46], *PALB2* breast = 6.16 [95% CI: 1.98–19.21], *CHEK2* breast = 4.89 [95% CI: 2.01–11.34]). Grade 3 ER-positive HER2-negative, grade 3 and triple negative (TN) tumors were enriched in cases with *PALB2* PGVs compared with all breast cancers known to our service (respectively: 15/43, 254/1,843, *P* = 0.0005; 28/37, 562/1,381, *P* = 0.0001; 12/43, 204/1,639, *P* < 0.0001). *PALB2*_PGV likelihood increased with increasing Manchester score (MS) (MS < 15 = 17/1,763, MS 20–39 = 11/520, *P* = 0.04) but not for *CHEK2*_1100delC (MS < 15 = 29/1,762, MS 20–39 = 4/520). *PALB2* PGVs showed perfect segregation in 20/20 first-degree relatives with breast cancer, compared with 7/13 for *CHEK2*_1100delC (*P* = 0.002).

**Conclusion:**

*PALB2* PGVs and *CHEK2*_1100delC together account for ~2.5% of familial breast/ovarian cancer risk. *PALB2* PGVs are associated with grade 3, TN, and grade 3 ER-positive HER2-negative breast tumors.

## INTRODUCTION

Breast cancer is the most common form of cancer in women.^1^ It has been known for over 30 years that breast cancer predisposition can be inherited in an autosomal dominant manner with around 4% of cases on a population basis being compatible with the inheritance of a high-risk (circa 50–80% lifetime risk) pathogenic germline gene variant (PGV).^2^ The *BRCA1* and *BRCA2* genes were identified as such genes in 1994 and 1995 accounting for around 2% of incident breast cancers and with a combined population prevalence of 1 in 300–400.^3^ Although *TP53* can cause a pattern consistent with dominantly inherited breast cancer it is usually associated with onset at extremely young ages, is rare (circa population prevalence 1 in 5,000) and more typically causes patterns of other malignancies, particularly sarcoma.^4^ Other extremely rare genes have been suggested as potential high-risk genes with lifetime risks of >40% such as *CDH1*, *PTEN*, and *STK11*, but definitive evidence based on case control data has been lacking due to their rarity (population prevalence ~1 in 50–100,000 for each).^5^ *PALB2* was originally identified in 2007 as a moderate risk gene conferring only around a 2.3-fold relative risk;^6^ this was, however, based on identification in 10/923 *BRCA1/2* negative breast cancer cases, but 0/1,083 controls. Therefore, the originally calculated relative risk was based on study of the families rather than the odds ratio (OR) generated from case–control analysis because this is usually inflated by using familial risk probands. Given the low predicted lifetime risk of only 20–30%, *PALB2* testing was not widely utilised until it became included in gene panel testing. It was not until more than 7 years later in 2014 that penetrance analysis in 158 families suggested risks approaching high-risk with a 35% (95% CI: 26–46%) estimated risk by age 70 years.^7^ This risk was higher in the context of a close relative with breast cancer. This analysis was updated recently in 524 affected families with estimated risks to age 80 years of 53% (95% CI: 44–63%) for female breast cancer and 5% (95% CI: 2–10%) for ovarian cancer.^8^

In contrast to *PALB2*, the original assessment of *CHEK2* as a moderate risk gene has been maintained from its discovery in 2002.^5,9–11^ Nonetheless, like *PALB2*, the risk increases when there is a positive family history of breast cancer.^11^ In the UK, while initially consensus was reached to include both *CHEK2* and *PALB2* in breast cancer gene panels in the UK,^12^ mainstreaming of tests to oncologists and other specialties in both breast and ovarian cancer now only includes *PALB2* in addition to *BRCA1* and *BRCA2.*^13^

In view of these changes in recommendations, namely the recent addition of *PALB2*, but not *CHEK2*, to UK genetic testing guidance, we report our single-center experience to date of germline *PALB2* and *CHEK2* c.1100delC; p.(Thr367MetfsTer15) (*CHEK2*_1100delC) testing in breast/ovarian cancer; these data will help provide evidence for future testing guidance.

## MATERIALS AND METHODS

### Patients

Women were eligible for this study if they had a histologically confirmed diagnosis of invasive or in situ breast cancer or an epithelial nonmucinous ovarian cancer and had undergone germline testing of *BRCA1*, *BRCA2*, *PALB2*, and *CHEK2*_c.1100delC for PGVs. Women were either referred for genetic testing to the Manchester Center for Genomic Medicine (MCGM) or the Family History Risk and Prevention Clinic (FHRPC) at the Nightingale Center, Wythenshawe Hospital (*n* = 2,603).^14^ In addition, 524 women were tested as part of the population based Predicting the Risk Of Cancer At Screening (PROCAS) study, in Greater Manchester.^15^ Demographic details of the study population are outlined in Table 1. Women without a breast cancer diagnosis (*n* = 1,567, aged 46–73 years), who were also recruited to the PROCAS study, were included as controls.

**Table 1:** Summary demographic features of individuals in cohort (*n* = 3,127).

**Breast cancers: PROCAS**	**Total tested**	***BRCA1/2***	***PALB2***	***CHEK2*_1100delC**
Lobular	45	0	0	0
Grade 1	69	0	0	0
IDC grade2 ERpos	134	1	0	2
IDC grade3 ERpos	56	3	1	1
TNT total	28	0	0	0
HER2+ total	31	0	0	0
DCIS total	80	2	1	2
Total with pathology	443	6	2	5
Invasive carcinomas, no pathology	81	3	2	4
**Total**	**524**	**9**	**4**	**9**
**Breast cancers: FHRPC/MCGM**	**Total tested**	***BRCA1/2***	***PALB2***	***CHEK2*_1100delC**
Lobular	220	9	3	2
Grade 1	164	3	1	3
IDC grade2 ERpositive	425	36	3	2
IDC grade3 ERpositive	329	39	7	9
TNT total	260	52	8	1
HER2positive total	146	4	2	2
DCIS total	132	15	1	3
Total with pathology	1,676	158	25	22
Invasive carcinomas, no pathology	677	36	4	13
**Total**	**2,353**	**194**	**29**	**35**
Breast cancers total (breast +/− ovary)	2,877	203	33	44
Ovarian cancer	302	32	2	1
Breast & ovarian cancer cases	52	7	0	0
**Overall total**	**3,127**	**228**	**35**	**44**
Bilateral breast	332	43	3	5
**Breast cancer age (years)**	**Total tested**	***BRCA1/2***	***PALB2***	***CHEK2*_1100delC**
≤30	214	12	4	3
31–40	501	69	5	9
41–50	968	74	10	13
51–60	764	38	8	13
61–70	358	11	5	3
>70	72	2	1	3
**Total**	**2,877**	**202**^**a**^	**33**	**44**

*DCIS* ductal carcinoma in situ, *FHRPC* Family History Risk and Prevention Clinic, *IDC* invasive ductal carcinoma, *MCGM* Manchester Center for Genomic Medicine, *PROCAS* Predicting the Risk Of Cancer At Screening study, *TNT* triple negative tumor.

^a^One age unknown.

For pathology comparisons we included 1,843 women known to the FHRPC/MCGM who had developed breast cancer but did not have a PGV in the known breast cancer predisposition genes. We also included women with a *PALB2* associated breast cancer in the extended families.

Clinical or research consent was given for extended testing of breast cancer associated genes (approval from the North Manchester Research Ethics Committee, reference 09/H1008/81 [PROCAS] and 08/H1006/77).

### Genetic screening

For women that were seen through the MCGM and FHRPC, DNA was extracted from lymphocytes, whereas those recruited through PROCAS (including the controls) had DNA extracted from saliva. Those attending MCGM had DNA initially analyzed for PGVs in *BRCA1/2* and *CHEK2*_c.1100delC (with the standard clinical panel expanded to include *PALB2* in 2016) by a combination of next-generation sequencing and multiplex ligation-dependent probe amplification (MLPA). Testing of DNA in the cohort and controls all patients was performed by a combination of targeted sequencing, panel test, and exomes (Supplementary Table 1).

Variants were classified according to the American College of Medical Genetics and Genomics/Association for Molecular Pathology (ACMG/AMP) guidelines.^16^ Variants that were classified as likely pathogenic and pathogenic only are reported here and combined along with gene rearrangements as PGVs.

Tumor pathology information was obtained for each case when available through hospital records, and cancer registries as previously described.^17^ The probability of a *BRCA1/2* PGV was determined using the Manchester score (MS) for each affected individual.^18^ This adds scores for each breast and ovarian cancer in a direct lineage with increasing scores for earlier age at onset and pathologies in the proband suggestive of *BRCA1* such as high-grade serous ovarian cancer and triple negative breast cancer (TNT). A MS of 15–19 and 20–24 roughly equate to a 10% and 20% likelihood threshold for *BRCA1/2* respectively with a score of ≥40 equivalent to a likelihood of >75%.^18^

Statistical analysis was undertaken using GraphPad Prism version 9.0.1 for Windows (GraphPad Software, San Diego, CA, USA) and GraphPad QuickCalcs.^19^

## RESULTS

### Pathogenic germline variants

A total of 3,127 index cases with breast and/or ovarian cancer have undergone testing for PGVs of *BRCA1*, *BRCA2*, *PALB2*, and *CHEK2*_c.1100delC (Table 2). There were 35 (1.12%) with PGVs in *PALB2* and 44 (1.41%) with *CHEK2_*c.1100delC. This compared to rates of 3/1,567 (0.19%, *P* = 0.0004) and 5/1,567 (0.32%, *P* = 0.0003) for *PALB2* and *CHEK2*_c.1100delC respectively in the PROCAS controls, generating ORs of 5.90 (95% CI = 1.92–18.36) and 44.46 (95% CI = 1.86–10.46) respectively.

**Table 2:** Samples tested, and number PGVs detected, for *PALB2*, *CHEK2_*1100delC, *BRCA1*, and *BRCA2* by source and Manchester score (MS).

MS	2–8	9–10	11–12	13–14	15–19	20–24	25–29	30–39	40+	Total
**FHx breast only**	301	235	300	296	534	253	106	68	15	2,108
*BRCA1/2*	3	9	6	14	38	35	23	24	6	158
*PALB2*	1	4	2	4	5	6	2	1	0	25
*CHEK2*_1100delC	3	2	6	9	9	3	0	0	0	32
**FHx breast/ovarian**	35	85	18	42	98	82	71	44	20	495
*BRCA1/2*	1	4	2	0	15	13	9	12	5	61
*PALB2*	1	1^a^	2	0	1^b^	0	1	0	0	6
*CHEK2*_1100delC	0	0	0	0	2	0	0	1	0	3
**PROCAS all**	432	24	25	15	14	7	3	4	0	524
*BRCA1/2*	5	0	0	2	0	1	0	1	0	9
*PALB2*	2	0	0	0	1	1	0	0	0	4
*CHEK2*_1100delC	9	0	0	0	0	0	0	0	0	9
**Total tested**	**768**	**344**	**343**	**353**	**646**	**342**	**180**	**116**	**35**	**3,127**
**Total*PALB2***	**4**	**5**	**4**	**4**	**7**	**7**	**3**	**1**	**0**	**35**
% *PALB2*	0.5%	1.5%	1.2%	1.1%	1.1%	2.1%	1.7%	0.9%	0.0%	1.1%
**Total*CHEK2*_1100delC**	**12**	**2**	**6**	**9**	**11**	**3**	**0**	**1**	**0**	**44**
% *CHEK2*	1.6%	0.6%	1.7%	2.5%	1.7%	0.9%	0%	0.9%	0.0%	1.4%
**Total*BRCA1/2***	**9**	**13**	**8**	**16**	**53**	**49**	**32**	**37**	**11**	**228**
% *BRCA1/2*	1.2%	3.8%	2.3%	4.5%	8.2%	14.3%	17.8%	31.9%	31.4%	7.3%
% *PALB2* in *BRCA1/2* negative	0.5%	1.5%	1.2%	1.2%	1.2%	2.4%	2.0%	1.3%	0.0%	1.2%
% *CHEK2*_1100delC in *BRCA1/2* negative	1.6%	0.6%	1.8%	2.7%	1.9%	1.0%	0.0%	1.3%	0.0%	1.5%

*PGV* pathogenic germline variant, *PROCAS* Predicting the Risk Of Cancer At Screening study, *FHx* family history.

^a^Individual with ovarian cancer only and no family history of breast/ovarian cancer.

^b^Individual with ovarian cancer only and family history of breast cancer.

Testing of 302 cases with ovarian cancer detected two *PALB2* PGVs, (c.2167_2168delAT; p.[Met723ValfsTer21] and c.3113G>A; (p.Trp1038Ter]) (OR = 3.47, 95% CI = 0.61–17.07, *P* = NS), and one with *CHEK2_*c.1100delC (OR = 1.04, 95% CI = 0.09–7.49, *P* = NS). Considering the breast cancer only index cases, there were 33 *PALB2* PGVs and 43 *CHEK2*_c.1100delC PGVs (*PALB2*: OR = 6.16, 95% CI = 1.98–19.21, *P* = 0.0003; *CHEK2*: OR = 4.83, 95% CI = 2.01–11.34, *P* = 0.0001). Testing in the population based PROCAS breast cancer study identified *PALB2* PGVs in 4/524 (0.76%) (OR = 4.01, 95% CI = 1.07–15.95, *P* = 0.071) and the *CHEK2*_c.1100delC PGV in 9/524 (1.71%) (OR = 5.46, 95% CI = 1.93–14.60, *P* = 0.0021), whereas testing of breast cancer index cases in the context of family history/early onset breast cancer only, detected *PALB2* PGVs in 29/2,301 (1.26%) (OR = 6.65, 95% CI = 2.26–20.86, *P* = 0.0002) and the *CHEK2* c.1100delC PGV in 34/2,301 (1.48%) (OR = 4.69, 95% CI = 1.88–11.12, *P* = 0.0002).

The mean age at diagnosis of first breast cancer for those with a *PALB2* PGV was 49.8 years (median 50 years, SEM = 2.30, range = 24–77 years) and, for the *CHEK2*_c.1100delCPGV, 49.3 years (median 49.3 years, SEM 1.83, range 27–76 years).

Overall, there were three common PGVs in *PALB2*. There were ten instances of c.3113G>A; p.(Trp1038Ter) in cases and one in controls, and four each of c.3116del; p.(Asn1039llefsTer2) and c.3549C>G; p.(Tyr1183Ter), with neither occurring in controls (Table 3). Together, these accounted for >50% (20/38) of the *PALB2* PGVs identified. Considering germline gene rearrangements and CNVs, three multiexon deletions and a translocation (46,XX,t[5;16][q33.1;p12.2]), of *PALB2* were detected in the patient cohort.

**Table 3:** Pathogenic variants in *PALB2* with breast pathology and receptor status and Manchester score (MS).

Patient^a^	First breast cancer age (years)	Grade	Tumor type	ER status	HER2 status	Second breast cancer age (years) and details where known	MS	*PALB2* PGV
1	67	2	IDC	Pos	Neg		14	c.1059_1077delinsGG; p.(Ser354GlyfsTer4)
2	54	3	IDC	NT	NT		14	c.1431del; p.(Ser478LeufsTer7)
3	24	3	IDC	Pos	Neg		12	c.1467_1468deI; p.(Pro490ArgfsTer5)
4	45	3	ILC	Pos	Neg		9	c.196C>T; p.(Gln66Ter)
5	39	3	ILC	Pos	Neg		26	c.2052del; p.(Arg686GlyfsTer23)
6	57	3	IDC	Neg^b^	Neg^b^		30	c.2325dup; p.(Phe776IlefsTer26)
7	77	3	IDC	Pos	Neg	77, grade 2, ERpos, HER2neg	23	c.2386G>T; p.(GIy796Ter)
8	50	3	IDC	Pos	Pos		11	c.2748+1G>A; p.?
9^c^	63	3	IDC	Pos	Neg		20	c.2982dup; p.(Ala995CysfsTer16)
10	55	3	IDC	Neg^b^	Neg^b^		10	c.3113G>A; p.(Trp1038Ter)
11	29	3	IDC	Pos	Neg		20	c.3113G>A; p.(Trp1038Ter)
12	28	Intermediate	DCIS	Pos	Neg		12	c.3113G>A; p.(Trp1038Ter)
13	45	3	IDC	Pos	Neg		17	c.3113G>A; p.(Trp1038Ter)
14	40	3	IDC	Neg^b^	Neg^b^		24	c.3113G>A; p.(Trp1038Ter)
15	46	3	IDC	Pos	Neg		9	c.3113G>A; p.(Trp1038Ter)
16	59	3	IDC	Neg^b^	Neg^b^		18	c.3113G>A; p.(Trp1038Ter)
17^c^	65	NK	IDC	NT	NT		8	c.3113G>A; p.(Trp1038Ter)
18^c^	63	NK	IDC	NT	NT		18	c.3113G>A; p.(Trp1038Ter)
19^c^	48	NK	DCIS	Pos	Neg		5	c.3113G>A; p.(Trp1038Ter)
20	54	3	IDC	Neg^b^	Neg^b^		14	c.3116del; p.(Asn1039llefsTer2)
21	55	NK	IDC	NT	NT		17	c.3116del; p.(Asn1039llefsTer2)
22	61	NK	IDC	NT	NT		9	c.3116del; p.(Asn1039llefsTer2)
23	64	NK	IDC	NT	NT	73	22	c.3116del; p.(Asn1039llefsTer2)
24	41	3	IDC	Neg^b^	Neg^b^		24	c.3256C>T; p.(Arg1086Ter)
25	39	2	IDC	Pos	Neg		13	c.3549C>G; p.(Try1183Ter)
26	68	2	IDC	Pos	Neg		25	c.3549C>G; p.(Tyr1183Ter)
27	38	NK	ILC	NT	NT		27	c.3549C>G; p.(Tyr1183Ter)
28	44	3	IDC	Pos	Pos		21	c.3549C>G; p.(Tyr1183Ter)
29	62	NK	IDC	NT	NT		16	c.786del; p.(Glu263AsnfsTer16)
30	38	3	IDC	Pos	Neg		5	^d^Deletion of exons 5–7
31	53	3	IDC	Neg^b^	Neg^b^		22	^d^Deletion of exons 8–10
32	44	1	IDC	Pos	Neg	46, ERneg, HER2pos	19	^d^Deletion of exons 8–10
33	27	3	IDC	Neg^b^	Neg^b^		17	46,XX,t(5;16)(q33.1;p12.2)

*DCIS* ductal carcinoma in situ, *IDC* invasive ductal carcinoma, *ILC* invasive lobular carcinoma, *NK* grade not known, *NT* not tested.

^a^None developed ovarian cancer.

^b^Tumors were triple negative (ER/PR/HER2).

^c^PROCAS participant.

^d^These deletions were detected by multiplex ligation-dependent probe amplification (MLPA) and so the precise breakpoints are not known. However, the exons either side of those deleted were shown to be present in each patient (exons 4 and 8 for patient 30, exons 7 and 11 for patients 31 and 32). Probe locations (hg18) for exons 4–11 are as follows: exon 4, chr16:23540210-23540280; exon 5, chr16:23541776-23541854; exon 6, chr16:23542867-23542956; exon 7, chr16:23545151-23545230; exon 8, chr16:23548027-23548102; exon 9: chr16:23549183-23549259; exon 10: chr16:23554181-23554263; exon 11: chr16:23556620-23556707.

### *PALB2* PGVs: receptor status and grade

Table 3 shows the MS, age, and pathology information for breast tumors where a germline *PALB2* PGV was detected. Where pathology grade and receptor status were known, 8/25 breast tumors were triple negative (all grade 3). This was similar to the numbers and age at diagnosis of TNTs  known to our service with a *BRCA2* PGV (*n* = 9) (*PALB2* PGV TNT: mean age 48.6 years, median 54.0 years, range 27–59 years; *BRCA2* PGV TNT: mean age 46.8 years, median 48 years, range 33–55 years), but significantly fewer than for a *BRCA1* PGV (*P*  < 0.0001). While there was a trend toward increasing age of TNT diagnoses with a *PALB2* PGV as compared with *BRCA1* PGV associated TNTs (*BRCA1* PGV TNT: mean = 40.4 years, median = 39 years, range = 22–77 years), this was not significant (*P* = 0.060).

To investigate the breast pathology where there is a *PALB2* PGV further, we then considered all the patients with *PALB2* associated breast cancers known to our service where a full histological record was available (*n* = 43), some of whom had genetic testing through other sources and so are not included in the 33 presented in Table 3. Here, the TNT phenotype occurred in 12/43 (27.9%) *PALB2* PGVs, as compared with 11.1% (204/1843) of all breast cancers cases known to our service where full histology was known (OR = 3.11, 95% CI = 1.61–5.96, *P* = 0.002).

We then investigated other breast cancer subtypes and whether they were also enriched in heterozygotes for a *PALB2* PGV. A grade 3 ER-positive HER2-negative phenotype occurred in 15/43 (34.9%) *PALB2* PGVs, while accounting for 254/1843 (13.8%) breast cancers (OR = 3.35, 95% CI = 1.76-6.44, *P* = 0.0005).

For a grade 3 phenotype, regardless of receptor status, these again were overrepresented occurring in 28/37 (75.7%) individuals with a *PALB2* PGVs as compared with 562/1381 (40.7%) of all invasive ductal carcinomas known to our service and testing negative for *BRCA1*, *BRCA2*, and *PALB2* PGVs (OR = 4.53, 95% CI = 2.11-9.65, *P* < 0.0001).

### Manchester score

To assess the probability of PGVs in *BRCA1/2*, MS was determined for all affected women (Table 2). While the likelihood of a *PALB2* PGV increased with increasing MS, no such trend was seen for *CHEK2*_1100delC. Overall, the rates of *CHEK2*_1100delC were similar in *BRCA1/2* negative cases at the lowest MS of <9 (12/759, 1.6%) compared with 4/520 with a MS = 20–39, 0.8%, *P* = NS) (Table 4). For *PALB2* there was a significantly higher likelihood of a PGV for MS of 20–39 than for MS < 9 and MS < 15 (*P* < 0.05) (Table 4).

**Table 4:** Proportion of *PALB2* and *CHEK2_*1100delC pathogenic germline variants (PGVs) by Manchester score (MS) in *BRCA1/2* negative families.

MS	Total	*BRCA1/2*	No *BRCA1/2*	*PALB2*		*CHEK2*	
				***N***	**%**	***N***	**%**
≤8	768	9	759	4	0.5%	12	1.6%
9–10	344	13	331	5	1.5%	2	0.6%
11–12	343	8	335	4	1.2%	6	1.8%
13–14	353	16	337	4	1.2%	9	2.7%
Total <15	**1,808**	**46**	**1,762**	**17**	**1.0%**	**29**	**1.6%**
20–24	342	49	293	7	2.4%	3	1.0%
25–29	180	32	148	3	2.0%	0	0.0%
30–39	116	37	79	1	1.3%	1	1.3%
40+	35	11	24	0	0.0%	0	0.0%
Total ≥20	**673**	**129**	**544**	**11**	**2.0%**	**4**	**0.7%**

*PALB2* MS (20–29) vs. MS (<9), *P* = 0.010. *PALB2* MS (20–29) vs. MS (<15), *P* = 0.048. *PALB2* MS (20–39) vs. MS (<9), *P* = 0.015. *PALB2* MS (20–39) vs. MS (<15), *P* = 0.043.

CHEK2_1100delC MS (20–29) vs. MS (<9), *P* = 0.280. CHEK2_1100delC MS (20–29) vs. MS (<15), *P* = 0.180. CHEK2_1100delC MS (20–39) vs. MS (<9), *P* = 0.30. CHEK2_1100delC MS (20–39) vs. MS (<15), *P* = 0.207.

### Segregation of PGVs with breast cancer in families

There was perfect segregation of *PALB2* PGVs in all 20 first-degree relatives (FDRs) with breast cancer who were available for testing; many FDRs with breast cancer were deceased and unavailable for testing and not all living FDRs opted for testing. This compared to only 7/13 for *CHEK2_*1100delC (*P* = 0.002) families (Table 5). Although there was only 88.8% and 87.4% segregation in *BRCA1* and *BRCA2* respectively, this was not significantly different to *PALB2*.

**Table 5:** Testing of first-degree relatives (FDRs) with breast cancer.

Gene	Number of FDRs with breast cancer	Number tested	PGV positive	PGV negative	Proportion positive (95% CIs)	Number untested	% Untested (95% CIs)
*BRCA1*	653	338	300	38	88.8% (84.9–91.7)	315	48.2% (53.1–44.2)
*BRCA2*	752	405	354	51	87.4% (83.8–90.3)	347	46.1% (42.6–49.7)
*PALB2*	31	20	20	0	100.0% (83.9–100.0)	11	35.5% (21.1–53.1)
*CHEK2_*1100delC	41	13	7	6	53.8% (29.1–76.8)	28	68.3% (53.0–80.4)

*CI* confidence interval, *PGV* pathogenic germline variant.

## DISCUSSION

This is the largest study looking at the prevalence of *PALB2* PGVs and the *CHEK2*_1100delC PGV from a single genetics center in patients attending with a personal diagnosis of breast and/or nonmucinous ovarian cancer. We demonstrated *PALB2* OR ≥ 6 and *CHEK2* OR ≥ 4 for breast and breast–ovarian cancer, and found specific breast cancer pathology associations where a *PALB2* PGV is present.

The timely importance of our study reflects the recent publication and implementation of the National Health Service England (NHSE) national test directory.^13^ In England, testing for only *BRCA1*, *BRCA2*, and *PALB2* PGVs is offered for hereditary breast cancer. Despite *CHEK2* and *ATM* also being recommended for inclusion by the UK Cancer Genetics Group,^12^ these genes have been omitted. Furthermore, recent publication of the BRIDGES case control study^20^ showed truncating variants of these five genes to be strongly associated (*P* < 0.0001) with breast cancer risk.

The association of germline *BRCA1* and *BRCA2* PGVs with high-risk breast cancer predisposition has been well recognized and clinical diagnostic testing of these genes has been offered for the past 23 years in Manchester. While *PALB2* was identified as a cause of hereditary breast cancer in 2007,^6^ it is only more recently that *PALB2* PGVs have been shown to be associated with a >50% lifetime breast cancer risk in females.^7,8^ Routine diagnostic testing of *PALB2* has been offered in MCGM since 2016, with some samples having been analyzed previously through research studies with diagnostic laboratory confirmation of any PGVs identified.

We detected an excess of *CHEK2*_1100delC and *PALB2* PGVs in our patients and this was significant across all subgroups, except for *PALB2* PGVs in the population based PROCAS subgroup. This is in keeping with PGVs of *PALB2* being relatively rare and associated with high-risk breast cancer predisposition, and so would be less likely to be detected in excess in our non-high-risk breast cancer cases.

Our analysis of MS and likelihood of a *PALB2* PGV showed that a higher MS (20–29 and 20–39) is strongly correlated with the presence of a germline *PALB2* PGV as compared with a lower MS (<15), the current NHSE threshold of diagnostic *BRCA1/2* and *PALB2* testing.^21^ Interestingly at very high MS, in our data set, the likelihood of a *PALB2* PGV appears to tail off as compared with *BRCA1/2*; further data will be needed to see if this finding is replicated. These data confirm the validity and clinical utility of the MS in the prediction of high-risk single gene breast cancer predisposition. Although the MS was designed for *BRCA1/2* PGV likelihood, as *PALB2* is a high-risk gene and associated with increasing frequency as MS rises, MS is also a useful marker for *PALB2* PGVs except at very high scores. The lack of association of MS with the *CHEK2*_1100delC PGV likely reflects the associated moderate risk breast cancer predisposition of *CHEK2*_1100delC. When working up families for breast cancer gene panel testing, our data does suggest that MS is not suitable for determining likelihood of the presence of the *CHEK2*_1100delC PGV.

We also detected two *PALB2* PGVs in individuals with high-grade serous ovarian cancer. *PALB2* PGVs are associated with a 5% lifetime risk of ovarian cancer^8^ and our data would suggest that the additional weighting within the MS for ovarian cancer (and not counting mucinous subtypes) is appropriate.

We confirmed the recently reported association of *PALB2* PGVs with a tendency to the development of TNTs,^22^ accounting for 28% of breast tumors occurring in female *PALB2* PGV heterozygotes known to our service. While this tumor phenotype is more typical of *BRCA1* PGVs, TNTs  occur in 16% of *BRCA2* associated breast cancers and at increasing ages as compared with *BRCA1.*^23^ In our data set, the mean age of the TNTs associated with a *PALB2* PGV (46.6 years) was similar to that for TNTs associated with a *BRCA2* PGV (46.8 years) and, while tending toward an older age than for *BRCA1* PGVs (40.4 years), this was not significant.

More striking was the association of grade 3 ER-positive HER2-negative tumors with a *PALB2* PGV, comprising 35% of breast cancers in our *PALB2* PGV cohort, which, to our knowledge, has not been previously demonstrated. While Hu et al.^22^ noted an OR of 5.2 for ER-positive HER2-negative breast tumors for *PALB2* PGVs, their study did not account for tumor grade. Considering grade in particular, we also found a marked excess of grade 3 tumors occurring in 76% of all *PALB2* PGV associated breast cancers. While our data may be biased toward referrals received where a woman has developed a high-grade breast cancer with or without a relevant family history, it is likely that such an association is real, although further data collection is needed to tease out these tumor phenotype correlations.

Our data would suggest that, for women who had a breast cancer where the histology is of a TNT or grade 3 ER-positive HER2-negative phenotype in association with a high MS, there may be merit it considering testing for germline *PALB2*_PGVs where *BRCA1/2* testing was negative.

The similarities of the phenotypes associated with *BRCA1*/*2* and *PALB2* PGVs, and strong association with MS, likely reflect the combined BRCA1-PALB2-BRCA2 functional unit that facilitates the repair of double stranded DNA breaks using the high-fidelity homologous recombination repair pathway.^24^

Exploitation of this HR pathway has enabled the utilization of poly (ADP-ribose) polymerase (PARP) inhibitors in the drug treatment of ovarian cancers associated with germline and/or somatic *BRCA1/2* PGVs and their investigation in clinical trials of advanced breast cancer. Given the functional interaction of PALB2 with BRCA1 and BRCA2, and the phenotypic similarities of the associated cancers presented within and by others,^8^ it is likely these agents will also be of utility in the treatment of PALB2 deficient cancers. In fact, a recent phase II trial investigating Olaparib in metastatic breast cancer showed an objective response rate of 82% where there was a germline *PALB2* PGV; furthermore, 85% of the breast cancers were ER-positive HER2-negative (grade not given).^25^

What is less clear is, despite BRCA1, BRCA2, and PALB2 forming a functional unit, why *BRCA1* and *BRCA2* PGVs are much more common than those affecting *PALB2*, with *BRCA1*/2 PGVs detected in 7% of our patient cohort and *PALB2* PGVs in 1%. While the reasons for these differing frequencies may not be clear, influencing factors may include the smaller size of *PALB2* and, until recently, *PALB2* PGVs have not been routinely screened for in either patient or population cohorts. Furthermore, *BRCA1/2* PGVs are thought to be as frequent as 1 in 250–300 of the general population.^26^

Of note we detected three recurrent PGVs of *PALB2* in our cohort, c.3113G>A; p.(Trp1038Ter), c.3116del; p.(Asn1039llefsTer2) and c.3549; p.(Tyr1183Ter), together accounting for 50% of the individuals with *PALB2* PGVs detected. These PGVs lie within the C-terminal WD40 domain of PALB2, which interacts with BRCA2. We note that for *PALB2* PGVs reported by Yang et al.,^8^ while PGVs were distributed throughout *PALB2*, c.3113G>A was the most common PGV identified in breast/ovarian cancer detected in 61 families, with c.3549C>G detected in 19 families, and c.3116del in 9. Given our much smaller data set we cannot prove or disprove the possibility of a founder effect in our local population in the absence of haplotype studies; however, it may be that these loci represent *PALB2* mutation hotspots, especially as the *PALB2* founder PGVs reported are not localized to the 3’ end.^27,28^

Regarding the *PALB2* del copy-number variants (CNVs) detected, both in this study and that reported by Yang et al.,^8^ again these all reside within the C-terminal WD40 domain that interacts with BRCA2. Thus, these data together may, in part, explain the similarities between the phenotypes seen with *BRCA2* and *PALB2* PGVs. Furthermore, it is likely that the genomic architecture toward the 3’ end of *PALB2* predisposes to recombination events given this is where the CNVs and the balanced translocation we detected have been described. We note that our *PALB2* testing strategy did not include CNVs for all our patients and so it is possible that further *PALB2* CNVs remain to be identified in this cohort. Based on the data we report, we would recommend that any clinical testing strategy for *PALB2* PGVs includes CNV analyses.

The association of *CHEK2* PGVs with moderate risk breast cancer predisposition is well recognized. CHEK2 has a role in DNA repair, although at an earlier stage whereby it detects then determines the cellular response to DNA damage.^29^ We detected the *CHEK2_*1100delC PGV in 44/3,127 cases compared with just 5/1,567 controls reflecting the also increased OR attained from the much larger BRIDGES study.^20^ There has been considerable debate regarding the utility of including the moderate risk penetrance genes, *CHEK2* and *ATM*, in breast cancer diagnostic genetic testing panels. The recent BRIDGES data would support their inclusion, given that, along with the high-risk breast cancer genes, *BRCA1*, *BRCA2*, and *PALB2*, truncating PGVs are associated with a significant increased breast cancer risk, all with OR > 2.^20^ Similar findings were reflected in the CARRIERS study^30^ although with an *ATM* OR < 2; possibly reflecting the classification of all variants identified, including missense, as being PGV only where classified as (likely) pathogenic in ClinVar.^31^ We do not have a comprehensive data set for *ATM* PGVs and therefore were not included in our analyses; but data from BRIDGES^20^ would suggest *ATM* ought to also be included in breast cancer diagnostic panels. For our future studies, we would seek to attain data for germline *ATM* PGVs and include in our comparisons.

Given truncating *CHEK2* PGVs have been associated with a relative risk of breast cancer of 2.2,^9^ a high risk of contralateral disease,^32^ and are enriched in this cohort and others, this would substantiate inclusion of testing for *CHEK2* PGVs in clinical diagnostic breast cancer genetic testing panels.

Considering segregation of *PALB2* PGVs and *CHEK2*_1100delC within a family, we detected perfect concordance for *PALB2* in FDR with breast cancer but not for *CHEK2* with only 7/13 testing positive for the familial variant. This likely reflects the moderate risk predisposition of *CHEK2_*1100delC and that breast cancer risk arises from the combined contribution of single PGVs and polygenic risk score (PRS) with PRS having a greater contribution where the single PGV effect is lower. Recently it has been shown that the combination of a high-risk breast cancer PRS and *CHEK2*_1100delC equated to a breast cancer lifetime risk equivalent to that of a *PALB2* PGV alone.^33^ These data suggest that *CHEK2*_1100delC should be incorporated into diagnostic breast cancer genetic testing panels as this knowledge of moderate risk PGVs, combined with a PRS, will become part of routine practice in breast cancer risk assessment.

In this single-center comprehensive study of germline *PALB2* PGVs and the *CHEK2*_1100delC PGV in breast/ovarian cancer, we show a 1% detection rate for *PALB2* PGVs, 1.4% for *CHEK2_*1100delC, and ~7% for *BRCA1*/*2* PGVs. Breast cancers associated with *PALB2* PGVs tended toward phenotypes seen with a *BRCA2* PGV, namely both triple negative and high-grade ER-positive HER2-negative tumors. The detection of *PALB2* and *CHEK2* PGVs, in addition to PGVs detected in *BRCA1*/*2*, in breast/ovarian cancer is important for accurate risk assessment and the activation of subsequent cancer prevention and early detection strategies in the individual and their family.

## Supplementary Information

Supplementary Information

## Data Availability

The data analyzed in this study are available from the corresponding author on request.

## References

[1] Ferlay J (2019). Estimating the global cancer incidence and mortality in 2018: GLOBOCAN sources and methods. Int. J. Cancer..

[2] Newman B (1988). Inheritance of human breast cancer: evidence for autosomal dominant transmission in high-risk families. Proc. Natl. Acad. Sci. U.S.A..

[3] Anglian Breast Cancer Study Group. (2000). Prevalence and penetrance of BRCA1 and BRCA2 mutations in a population-based series of breast cancer cases. Br. J. Cancer..

[4] Lalloo F (2003). Prediction of pathogenic mutations in patients with early-onset breast cancer by family history. Lancet..

[5] Easton DF (2015). Gene-panel sequencing and the prediction of breast-cancer risk. N. Engl. J. Med..

[6] Rahman N (2007). PALB2, which encodes a BRCA2-interacting protein, is a breast cancer susceptibility gene. Nat. Genet..

[7] Antoniou AC (2014). Breast-cancer risk in families with mutations in PALB2. N. Engl. J. Med..

[8] Yang X (2020). Cancer risks associated with germline PALB2 pathogenic variants: an international study of 524 families. J. Clin. Oncol..

[9] Meijers-Heijboer, H. et al. Low-penetrance susceptibility to breast cancer due to CHEK2(*)1100delC in noncarriers of BRCA1 or BRCA2 mutations. *Nat. Genet*. **31**, 55–59 (2002). 10.1038/ng879 11967536

[10] Schutte M (2003). Variants in CHEK2 other than 1100delC do not make a major contribution to breast cancer susceptibility. Am. J. Hum. Genet..

[11] Weidner AE (2020). Breast cancer screening implications of risk modeling among female relatives of ATM and CHEK2 carriers. Cancer..

[12] Taylor A (2018). Consensus for genes to be included on cancer panel tests offered by UK genetics services: guidelines of the UK Cancer Genetics Group. J. Med. Genet..

[13] NHS England. National Genomic Test Directory. Testing criteria for rare and inherited disease. https://www.england.nhs.uk/wp-content/uploads/2018/08/Rare-and-Inherited-Disease-Eligibility-Criteria-November-2020-21.pdf (2020).

[14] Howell A (2020). Long-term evaluation of women referred to a breast cancer family history clinic (Manchester UK 1987–2020). Cancers (Basel)..

[15] Evans, D. G. et al. Improvement in risk prediction, early detection and prevention of breast cancer in the NHS Breast Screening Programme and family history clinics: a dual cohort study. (Southampton, UK, NIHR Journals Library, 2016). 27559559

[16] Richards S (2015). Standards and guidelines for the interpretation of sequence variants: a joint consensus recommendation of the American College of Medical Genetics and Genomics and the Association for Molecular Pathology. Genet. Med..

[17] Moran A (2012). Risk of cancer other than breast or ovarian in individuals with BRCA1 and BRCA2 mutations. Fam. Cancer..

[18] Evans DG (2009). Addition of pathology and biomarker information significantly improves the performance of the Manchester scoring system for BRCA1 and BRCA2 testing. J. Med. Genet..

[19] GraphPad. https://www.graphpad.com/quickcalcs/ (2021).

[20] Breast Cancer Association Consortium, Dorling L, Carvalho S et al. (2021). Breast cancer risk genes—association analysis of rare coding variants in 34 genes in 60,466 cases and 53,461 controls. N. Engl. J. Med..

[21] The National Institute for Health and Care Excellence. Clinical guideline. Familial breast cancer: classification and care of people at risk of familial breast cancer and management of breast cancer and related risks in people with a family history of breast cancer. https://www.nice.org.uk/guidance/cg164/evidence/full-guideline-pdf-190130941 (2021). 31940157

[22] Hu C (2020). The contribution of germline predisposition gene mutations to clinical subtypes of invasive breast cancer from a clinical genetic testing cohort. J. Natl. Cancer Inst..

[23] Mavaddat N (2012). Pathology of breast and ovarian cancers among BRCA1 and BRCA2 mutation carriers: results from the Consortium of Investigators of Modifiers of BRCA1/2 (CIMBA). Cancer Epidemiol. Biomarkers Prev..

[24] Ducy M (2019). The tumor suppressor PALB2: inside out. Trends Biochem. Sci..

[25] Tung NM (2020). TBCRC 048: phase II study of olaparib for metastatic breast cancer and mutations in homologous recombination-related genes. J. Clin. Oncol..

[26] Grzymski JJ (2020). Population genetic screening efficiently identifies carriers of autosomal dominant diseases. Nat. Med..

[27] Behl S (2020). Founder BRCA1/BRCA2/PALB2 pathogenic variants in French-Canadian breast cancer cases and controls. Sci. Rep..

[28] Vagena A (2019). PALB2 c. 2257C>T truncating variant is a Greek founder and is associated with high breast cancer risk. J. Hum. Genet..

[29] Tung N, Silver DP (2011). Chek2 DNA damage response pathway and inherited breast cancer risk. J. Clin. Oncol..

[30] Hu C (2021). A population-based study of genes previously implicated in breast cancer. N. Engl. J. Med..

[31] National Center for Biotechnology Information. ClinVar. https://www.ncbi.nlm.nih.gov/clinvar/ (2021).

[32] de Bock GH (2004). Tumour characteristics and prognosis of breast cancer patients carrying the germline CHEK2*1100delC variant. J. Med. Genet..

[33] Mars N (2020). The role of polygenic risk and susceptibility genes in breast cancer over the course of life. Nat. Commun..

