# Supplementary material for: Clinical utility of testing for PALB2 and CHEK2 c.1100delC in breast and ovarian cancer
Source: Genet Med. 2021 Jun 10;23(10):1969–76. doi: 10.1038/s41436-021-01234-6 (PMC8486655; doi:10.1038/s41436-021-01234-6)
Supplement: Supplementary file 1 — Supplementary Information [file 41436_2021_1234_MOESM1_ESM.docx]

**Supplementary Information**

**Supplementary Table 1. Details of numbers of patients undergoing testing for *PALB2* and *CHEK2* c.1100delC PGVs and method used.**

| **Grouping** | **Method of Screening** | **Number of Samples** | **Number with *PALB2* PGV** | **Number with *CHEK2* c.1100delC** |
| --- | --- | --- | --- | --- |
| Familial Breast Cancer Study [1, 2] | Targeted sequencing of candidate genes^a^ | 850 | 6 | 21 |
| MCGM | MCGM specific testing  (*BRCA1/2*, *PALB2*)^b^ | 773 | 5 | 5 |
| PROCAS | BRIDGES [3] panel^a^ | 524 | 4 | 9 |
| Breast cancer panel test | Panel test | 514 | 12 | 6 |
| FHrisk | BRIDGES [3] panel^a^ | 249 | 4 | 1 |
| BGI | Exome | 217 | 4 | 2 |
| **Total** |  | **3127** | **35** | **44** |

^a^not include CNV analysis of *PALB2*

^b^includes MLPA analysis to detect deletions/duplications of these genes. The *BRCA2* MLPA test (MRC-Holland SALSA MLPA probemix P045) also detects the *CHEK2* c.1100delC PGV.

**Supplementary References**

1. Rahman N, Seal S, Thompson D, et al. PALB2, which encodes a BRCA2-interacting protein, is a breast cancer susceptibility gene. Nat Genet. 2007;39:165-167.
2. Schutte M, Seal S, Barfoot R, et al. Variants in CHEK2 other than 1100delC do not make a major contribution to breast cancer susceptibility. Am J Hum Genet. 2003;72:1023-1028.
3. Breast Cancer Association Consortium, Dorling L, Carvalho S, et al. Breast cancer risk genes - Association analysis of rare coding variants in 34 genes in 60,466 cases and 53,461 controls. N Engl J Med. 2021;384:428-439.
